# Adoptive transfer of dendritic cells expressing CD11c reduces the immunological response associated with experimental colitis in BALB/c mice

**DOI:** 10.1371/journal.pone.0196994

**Published:** 2018-05-08

**Authors:** Lisiery N. Paiatto, Fernanda G. D. Silva, Áureo T. Yamada, Wirla M. S. C. Tamashiro, Patricia U. Simioni

**Affiliations:** 1 Institute of Biosciences, Universidade Estadual Paulista, UNESP, Rio Claro, SP, Brazil; 2 Faculty of Food Engineering, University of Campinas, UNICAMP, Campinas, São Paulo, Brazil; 3 Department of Food, School of Nutrition, Federal University of Ouro Preto, Ouro Preto, Minas Gerais, Brazil; 4 Department of Biochemistry and Tissue Biology, Institute of Biology, University of Campinas, UNICAMP, Campinas, São Paulo, Brazil; 5 Department of Genetics, Evolution, Microbiology and Immunology, Institute of Biology, University of Campinas, UNICAMP, Campinas, São Paulo, Brazil; 6 Department of Biomedical Science, Faculty of Americana, FAM, Americana, São Paulo, Brazil; "INSERM", FRANCE

## Abstract

**Introduction:**

In addition to conventional therapies, several new strategies have been proposed for modulating autoimmune diseases, including the adoptive transfer of immunological cells. In this context, dendritic cells (DCs) appear to be one of the most promising treatments for autoimmune disorders. The present study aimed to evaluate the effects of adoptive transfer of DCs obtained from both naïve and ovalbumin (OVA)-tolerant mice on the severity of TNBS induced colitis and analyze the eventual protective mechanisms.

**Methods and results:**

To induce oral tolerance, BALB/c mice were fed 4mg/mL OVA solution for seven consecutive days. Spleen DCs were isolated from tolerant (tDC) and naïve (nDC) mice, and then adoptively transferred to syngeneic mice. Three days later, colitis was induced in DC treated mice by intrarectal instillation of 100μg2,4,6-trinitrobenzenesulfonic acid (TNBS) dissolved in 50% ethanol. Control subjects received only intrarectal instillation of either TNBS solution or a vehicle. Five days later, mice from all groups were euthanized and examined for physiological and immunological parameters. Regarding the phenotype, we observed that the frequencies of CD11^+^ MHC II^+^ and CD11^+^ MHCII^+^ CD86^+^ cells were significantly lower in DCs isolated from tolerant mice than in those from naive mice. However, pretreatment with both types of DCs was able to significantly reduce clinical signs of colitis such as diarrhea, rectal prolapse, bleeding, and cachexia, although only treatment with tDCs was able to prevent weight loss from instillation of TNBS. *In vitro* proliferation of spleen cells from mice treated with either type of DCs was significantly lower than that observed in splenic cell cultures of naïve mice. Although no significant difference was observed in the frequencies of Treg cells in the experimental groups, the frequency of Th17^+^CD4^+^cellsand the secretion of IL-17 were more reduced in the cultures of spleen cells from mice treated with either type of DCs. The levels of IL-9 and IFN-γ were lower in supernatants of cells from mice treated with nDCs.

**Conclusion:**

The results allow us to conclude that the adoptive transfer of cells expressing CD11c is able to reduce the clinical and immunological signs of drug-induced colitis. Adoptive transfer of CD11c^+^DC isolated from both naive and tolerant mice altered the proliferative and T cell responses. To the best of our knowledge, there is no previously published data showing the protective effects of DCs from naïve or tolerant mice in the treatment of colitis.

## Introduction

Imbalance of the intestinal microbiota as well as genetic and environmental factors can trigger chronic inflammation in the gastrointestinal tract, called inflammatory bowel diseases (IBD). Ulcerative colitis (UC) and Crohn's disease (CD) are the main IBD´s in humans. The pathophysiological mechanisms of IBD, however, are not yet fully understood. Thus, experimental models that mimic this disease in humans are important tools for studying these types of diseases[1–4].

Experimental models of colitis are being tested in order to develop new therapeutic agents including chemical induction, immune cell transfer, or genetic manipulation of laboratory animals [4–10]. Among the experimental models most used in the study of colitis, the intrarectal administration of TNBS in mice stands out for inducing signs and symptoms very similar to those observed in humans [11–13].

Mechanisms enrolled in the physiopathology of IBD include unbalancing cytokines such as interleukin (IL)-9, IL-10, IL-35, transforming growth factor (TGF)-β as well as enzymes, cell receptors, and transcription factors such as indolemine-2,3-dioxygenase(IDO), Cytotoxic T-Lymphocyte Antigen (CTLA)-4, Leukocyte Activation Gene (LAG) -3, perforin/antagonists and Glucocorticoid-Induced Tumor Necrosis Factor Receptor (GITR)) [14–18]. Several new therapies are under evaluation to treat and reduce the signs and progression of IBDs. Among the options for conventional treatments, adoptive transfer of tolerogenic cells such as DC and Treg cells emerges as a promising alternative under evaluation [19, 20].

Dendritic cells (DCs) are the major antigen presenting cells (APC) that play a relevant role in the activation of naive T lymphocytes [7, 21]. The DCs that inhabit the gastrointestinal tract, however, have a proven involvement in the modulation of peripheral tolerance, through the secretion of anti-inflammatory cytokines and stimulation of Treg cells [22–25]. Studies from our group and others suggest that oral tolerance can generate DCs with tolerogenic profile in peripheral lymphoid organs, thus leading to the increase of regulatory T cells [19, 26].

The aim of this study is to evaluate the effects of adoptive transfer of CD11c^+^ dendritic cells obtained from OVA-tolerant and naïve BALB/ c mice in experimental colitis induced by TNBS in syngeneic mice.

## Material and methods

### Animals

BALB/c female mice (20–25 g) at four weeks of age were obtained from the Multidisciplinary Center for Biological Research (CEMIB) of the University of Campinas (UNICAMP), Campinas, SP, Brazil. They were maintained in a specific pathogen-free environment at 25^o^ C ±1 under photoperiod of 12/12 hours. The mice were fed an autoclaved Nuvilab CR-diet and water *ad libitum* for 2–4 weeks before being used in experiments. The methods described in this manuscript were carried out in accordance with the ‘Guide for the Care and Use of Laboratory Animals’, as promoted by the Brazilian College of Animal Experimentation (COBEA), and was approved by the Ethics Committee for Animal Experimentation at the University of Campinas (CEUA/UNICAMP. Protocol #3077–1). All experimental procedures were performed under proper anesthesia and all efforts were made to minimize animal suffering. The experimental groups consisted of at least five animals. The general health of the mice was monitored daily for signs of inflammation such as rectal swelling, rectal bleeding, soft stool, or weight loss.

### Oral tolerance

The induction of oral tolerance to OVA was performed as described previously [26, 27]. Briefly, 4mg/mL OVA (Rhoster Commerce and Industry Ltda, Vargem Grande Paulista, SP, Brazil) was added to the water supply of BALB/c mice for 7 consecutive days. The control mice received protein-free water.

### Isolation of DCs

Dendritic cells (DCs) were isolated from the spleens of the OVA tolerant (tDC) or naïve (nDC) mice as previously described [20,28]. To perform DC isolation, spleen cells were incubated with magnetic beads coated with anti-CD11c antibodies (Clone 130-052-001, Miltenyi Biotec, Germany) and using column MS MACS (130-042-201, Miltenyi Biotec) with Mini MACS Separation Unit (421–02, Miltenyi Biotec), according to the manufacturer's recommendations.

### Phenotypic profile of DCs

After immunomagnetic separation, the mean yield in three independent experiments was 5.8% of the total cells present in the spleen suspensions. The population of isolated cells averaged 97% of CD11c^+^ cells, of which 63% were MHCII^+^. The phenotypic profiles of DCs were evaluated by flow cytometry, by labeling with anti-mouse CD11c - APC (Clone: HL3), anti-mouse MHC-II-PE (130-091-368, Miltenyi Biotec), anti-mouseCD80-FITC (Clone: 16-10A1), anti-mouseCD86-FITC (Clone: GL1), and anti-mouseCD40-FITC (Clone: 3/23) according to the manufacturer's instructions (BD Bioscience, USA). The readings were performed using the FACSCalibur (Becton-Dickinson, Franklin Lakes, NJ, USA) flow cytometer located at the Institute of Biology of UNICAMP, using FCS Express 5 Plus, Research Edition software.

### Syngeneic adoptive transfer of DCs

Three doses of 3x105 tDCs isolated from OVA tolerant BALB/c mice or nDCs isolated from naïve BALB/c mice were injected intravenously into naive syngeneic mice on days -5, -3, and +1 of TNBS intrarectal instillation, as previously described [19, 27, 28].

### Induction of experimental colitis

**Fig 1:**
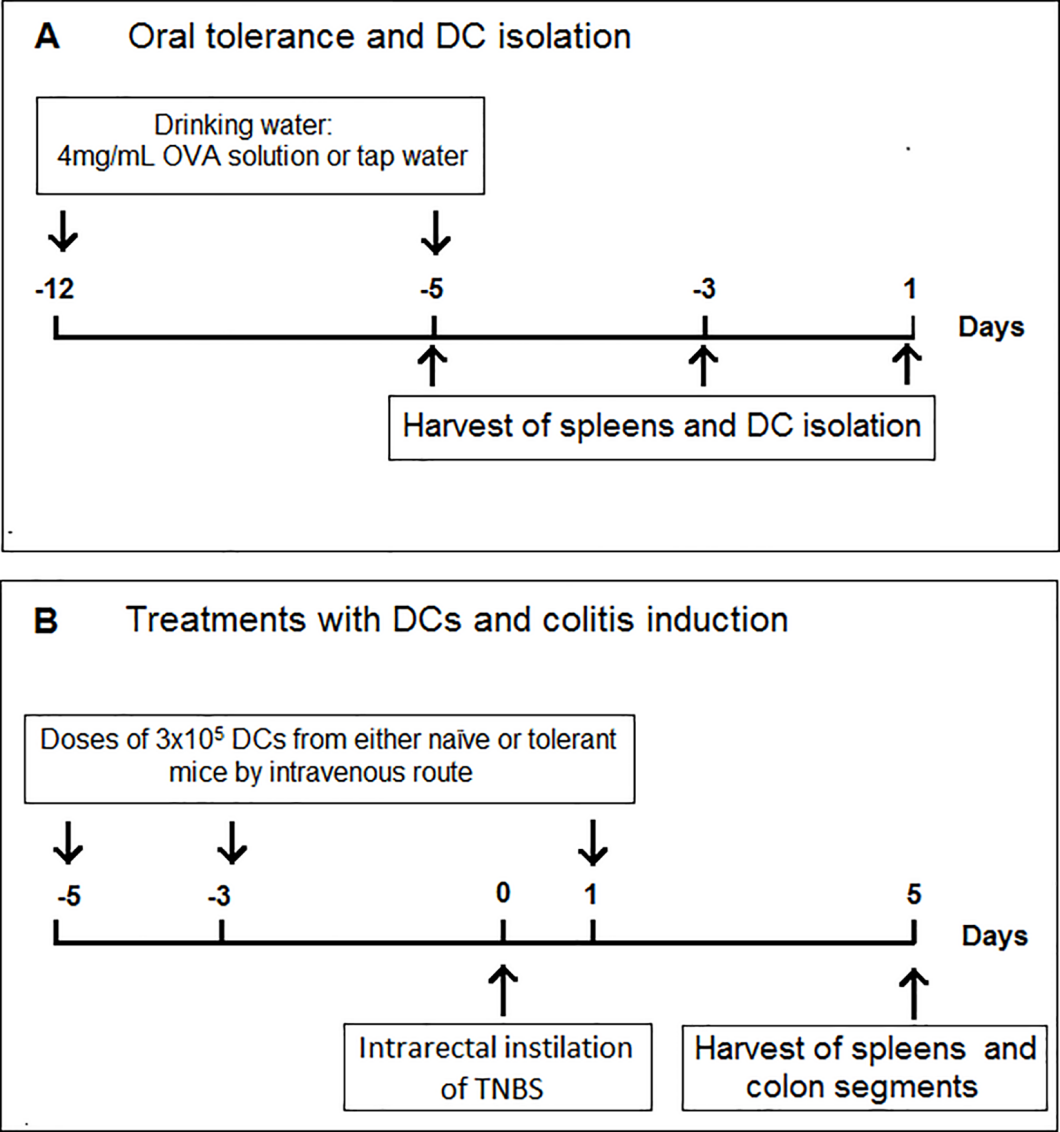
Flow chart of the adoptive transfer of dendritic cells and TNB-induced colitis. (Panel A) Naïve BALB/c mice were randomly divided into two groups (n = 10 per group), one of which was fed 4 mg/mL OVA solution for seven consecutive days (tolerant mice) and the other received protein-free water (normal mice). Mice from both groups were euthanized on days 1, 3, and 5 after discontinuation of the oral treatment and their splenic DCs were isolated using magnetic beads coated with anti-CD11c. (Panel B) Naïve BALB/c mice were randomly divided into four groups (n = 6 per group). Mice of two groups were injected intravenously with three doses of 3x105 DCs from tolerant or normal mice on days 5 and 3 prior to and 1 day after the induction of colitis. Twenty-four hours after the last injection, 100μL of 1 mg/mL TNBS solution was instilled intrarectally in DC-treated mice. The other two groups consisted of untreated mice (naïve mice) and mice treated with TNBS alone (colitic mice).

Colitis was induced by intrarectal administration of a single dose of 2,4,6-trinitrobenzenesulphonic acid (TNBS), as described elsewhere [29, 30], with modifications. Briefly, mice were anesthetized and instilled with 100μL of 1.0mg/mL TNBS (2,4,6—trinitrobenzenesulfonic acid; Sigma, USA) dissolved in 50% ethanol into the lumen of the colon. To ensure the agent entered the entire colon, mice were held in a vertical position for 30 s. Two control groups of mice that did not receive dendritic cells were used: 1) animals inoculated intrarectally with 100μL 50% ethanol; 2) animals inoculated intrarectally with 100μL TNBS dissolved in 50% ethanol (Fig 1).

### Evaluation of clinical symptoms of TNBS induced colitis

Animals in all groups were weighed daily until euthanasia on the fifth day after the induction of colitis. Weight variation was calculated as percentage. Clinical symptoms such as diarrhea, rectal prolapse, bleeding, and cachexia were registered and assigned as scores, ranging from 0 to 2, with 0: no change, 1: slight change, and 2: severe change [26].

### Histological analysis of colitis

Mice were euthanized, and the distal portions of the large intestines were removed. Two colon segments of 1 cm each were taken 4 cm from the anus, fixed in 4% buffered formalin, dehydrated with a grade ethanol solution, and embedded in paraffin (Paraplast Plus Sigma P3683). Slices of 5μm were obtained by microtome (Leica—model Jung Biocut 2035), mounted on clean glass slides, deparaffinized, rehydrated, and dyed with hematoxylin and eosin (Merck). Sections were evaluated for the presence of folds, hemorrhage, mucosal dilatation, and infiltration of leukocytes in two distal portions (from 1 to 2 cm and from 2 to 3 cm from the rectum) of the intestines.

Scores were determined by the sum of the values attributed to the presence of mucosal folds (from 0 to 5, 0—normal and evident folds; 1—slightly deformed folds; 2—deformed folds; 3—folds with impaired shape; 4—drastic reduction of folds and large infiltration; 5—nonexistence of folds and high tissue compromise), hemorrhage (0- none; 1-hemorrhage present; 2-large hemorrhage), mucosal dilatation (0—none or reduced voids; 1 apparent voids; 2—high voids), and lymphocytic infiltrate in the mucosa, submucosa and mesentery (0- none; 1- reduced infiltrate; 2 considerable infiltration with disorganization of the submucosa; 3—intense infiltrate). The results obtained in each group are presented as the mean ±S.E.M. The thickening of the wall of the colon was measured in micrometers in distal portions, with the Infinity Analyze Nikon H600L program (100X)[29, 30].

### Spleen cell proliferation

Mouse spleens from all experimental groups were collected aseptically, individually macerated, suspended in a lysis buffer, and pelleted by centrifugation at 200 g for 10 min. Cell concentration was adjusted to 1x10^6^ cells/mL in complete medium (RPMI1640 medium, Sigma, USA) supplemented with 10% fetal bovine serum (Cultilab, Campinas, Brazil). After washing, spleen cell suspensions were incubated with 25μM carboxyfluoresceinsuccinimidyl probe ester (CFSE) in complete medium at room temperature for 5 min, according to the manufacturer’s recommendations (Invitrogen, USA). Cells were then pelleted by centrifugation and suspended in complete medium. To determine the maximum uptake of CFSE, aliquots of the cell suspensions were fixed with 1% formaldehyde in PBS and analyzed by flow cytometer. CFSE-labeled cells were seeded into 96-well plates (Corning), in sextuplicate, and incubated in the presence of 2.5μg/mL ConA for 72 hours at 37°C. Cell cultures in absence of stimuli were used as the control.

The proliferation of T lymphocytes in cultures was assessed by the gate of CD4^+^CFSE^+^ cells. Acquisitions were performed with a FACSCalibur flow cytometer (FACSCalibur flow cytometer, BD Becton Dickinson, San Jose, CA) [20]. The results were analyzed with the FCS Express Plus Research Edition software (FCS Express Launcher). Results were expressed as proliferation index (fold change), as calculated in comparison to the control group [26]. Cells not stained with CFSE were also cultured in the presence of ConA and supernatants from spleen cell cultures of all experimental and respective control groups were collected for dosage of cytokines.

### Phenotypic profile of T-cells

The frequencies of TCD4^+^CD25^+^ Foxp3^+^ (Treg cells), TCD4^+^IL17^+^, TCD4^+^IFNγ^+^ and TCD4^+^IL-10^+^ cells in the cultures were assessed by a flow cytometer. Briefly, cell suspensions were washed and initially stained with anti-CD3 APC (clone 145-2C11, BD #553066), anti-CD4-PE (Clone GK1.5), and anti-CD25-FITC (Clone 7D4). Then, cells were permeabilized by the addition of a fixation/permeabilization buffer (Cytofix/Cytoperm fixation/permeabilization kit, Becton-Dickinson, BD). Suspensions were stained with anti-Foxp3-APC (clone FJK-16S), anti-IL-17-APC (clone eBIO17B7) or Alexa Fluor 647 (Clone TC11-18410), anti-IFN-γ-APC (Clone XMG1.2), and IL-10-APC (Clone JESS-16E3), 647 (Clone Q21-378), according to the manufacturer’s instructions. Acquisitions were performed with a FACScalibur flow cytometer and analyses were done with the FCS Express 5 Plus, Research Edition software [26].

### Determination of Th1, Th2, Th17 and Th9 cytokines

IL-2, IL-4, IL-6, IL-10, IL-17A, IFN-γ, and TNF-α were quantified in cultured supernatants of spleen cells by a flow cytometer, using a Multiplex CBA kit (BD Cytometric Bead Array Th1/Th2/Th17, San Diego, USA) according to the manufacturer’s instructions. Cells were acquired with a FACSCalibur cytometer and analyzed with FCAP Array TM Software Version 3.0 (BD). IL-9 determination was assayed with CBA flex set (BD Cytometric Flex Set Th9, San Diego, USA).

### Statistical analysis

A statistical analysis was performed using GraphPad Prism 5 (GraphPad Software, San Diego, CA, USA). The statistical significance of differences between the control and experimental groups were determined by one-way ANOVA, followed by Bonferroni’s test for multiple comparisons. The results were expressed as mean ± Standard Error of the Mean (S.E.M). Values were considered significant at p< 0.05. All data presented are representative of at least three independent experiments.

## Results and discussion

The self-destructive immune response observed in IBD correlates positively with the loss of microbiota tolerance, the activation of immune cells, and the presence of intense local inflammatory infiltrate. Although the mechanisms triggering the disease are not well understood, the presentation of enteric microorganism antigens by dendritic cells located in the lamina propria of the intestinal mucosa and in Peyer's plaques seems to represent a preponderant role in the etiology of these diseases [24, 31–33].

In previous studies carried out by our group, we hypothesized that the establishment of oral tolerance by ingestion of dietary proteins could restore, at least in part, the tolerogenic environment of the intestinal mucosa, with important reflexes to the systemic immune response. In this regard, we have previously shown that induction of oral tolerance to ovalbumin was able to prevent antigen-induced arthritis as well as TNBS-induced colitis in BALB/c mice [19, 26]. We have also shown that adoptive transfer of DCs from syngeneic OVA-tolerant animals could prevent arthritis induced by administration of OVA emulsified in complete Freund's adjuvant in BALB/c mice [19].

The intrarectal administration of 2,4,6-trinitrobenzenesulfonic acid (TNBS) is a protocol well established in studies of Crohn’s disease, since it closely mimics this human disorder [26, 34–37]. In the present study, we evaluated the effects of adoptive transfer of CD11c^+^DCs collected from naive and OVA-tolerant mice on the course of TNBS-induced colitis in syngeneic animals, as well as on the *ex vivo* response of their splenic immune cells.

**Fig 2:**
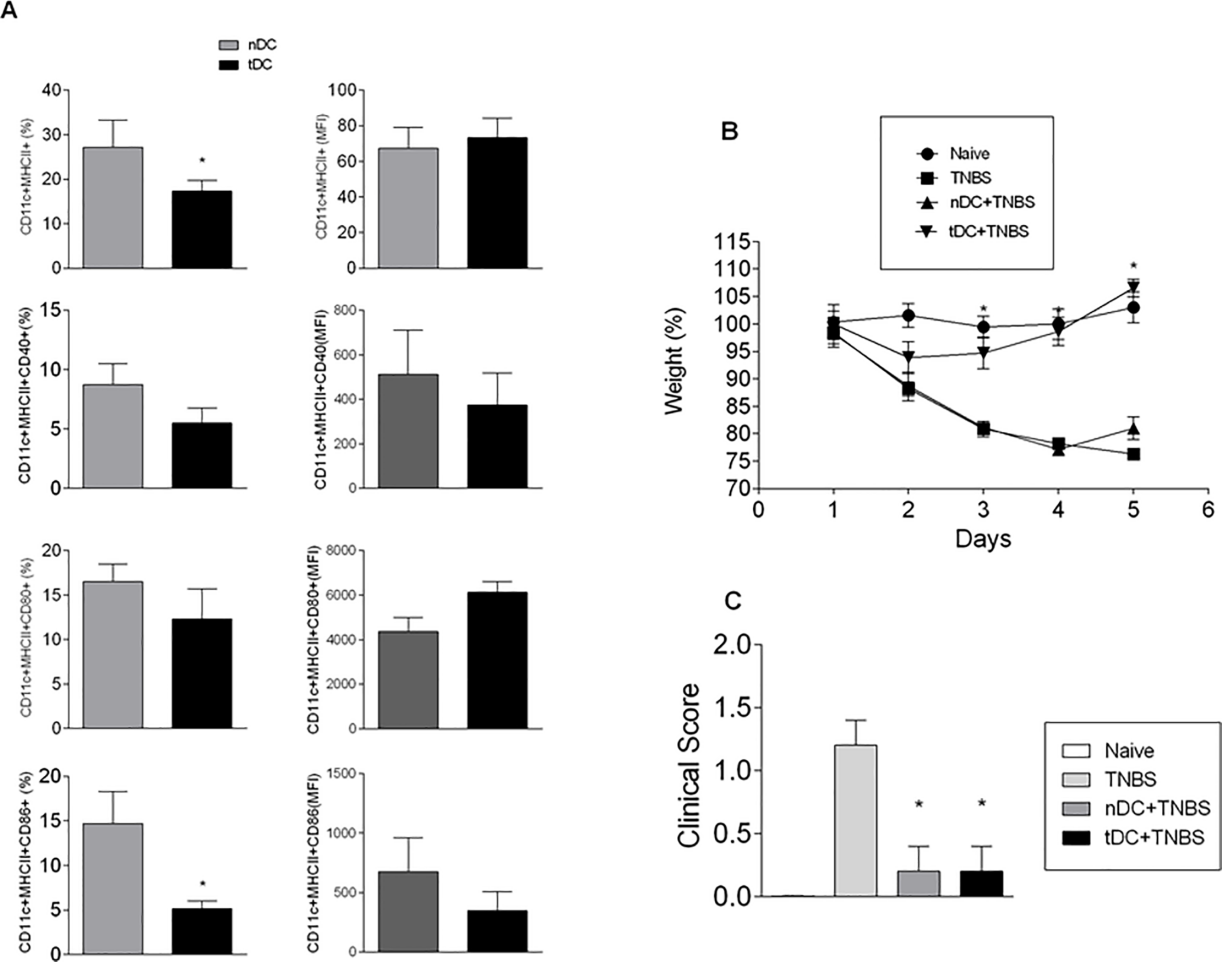
Adoptive transfer of CD11c^+^ dendritic cells isolated from OVA-tolerant mice reduces severity of experimental colitis. (Panel A) Characterization of DCs isolated from spleens of naive and OVA treated mice (nDC and tDC, respectively): The frequency of the CD40^+^, CD80^+^, and CD86^+^CD11c^+^ MHCII^+^ cells in DC preparations and the fluorescence intensity (MFI) geometric means of these markers were assessed by flow cytometry. (Panel B) Temporal changes in body weight: body weight was taken on a daily basis from 1 to 5 days after TNBS instillation and is represented as mean percentage in relation to the mean initial value (100%). (Panel C) Clinical signs: Clinical signs of colitis were evaluated for the presence of diarrhea, rectal prolapse, bleeding, and cachexia, assigning a score ranging from 0 to 2, with 0: no change, 1: slight change, and 2: severe change. Data are expressed as mean and S.E.M. (n = 5) and are representative of three independent experiments. ANOVA followed by Bonferroni post-test were used to determine statistical significance (p <0.05).

As depicted in Fig 2 (Panel A), prior treatment with oral OVA was able to reduce the percentage of DCs expressing CD86 surface marker compared to DCs from untreated mice, confirming data that we obtained previously [19]. Body weight variation and clinical signs, including cachexia, rectal prolapse, diarrhea, and bleeding, were analyzed on a daily basis for five days after TNBS instillation. As illustrated in Fig 2B, the weight loss associated with TNBS-induced colitis was prevented after adoptive transfer of CD11c^+^DCs from tolerant mice (tDC), but not after adoptive transfer of CD11c^+^DCs from naïve mice. Likewise, there was a significant reduction of the clinical signs associated with TNBS-induced colitis in mice treated with either tDC or nDC in comparison to the control group (Fig 2C).

**Fig 3:**
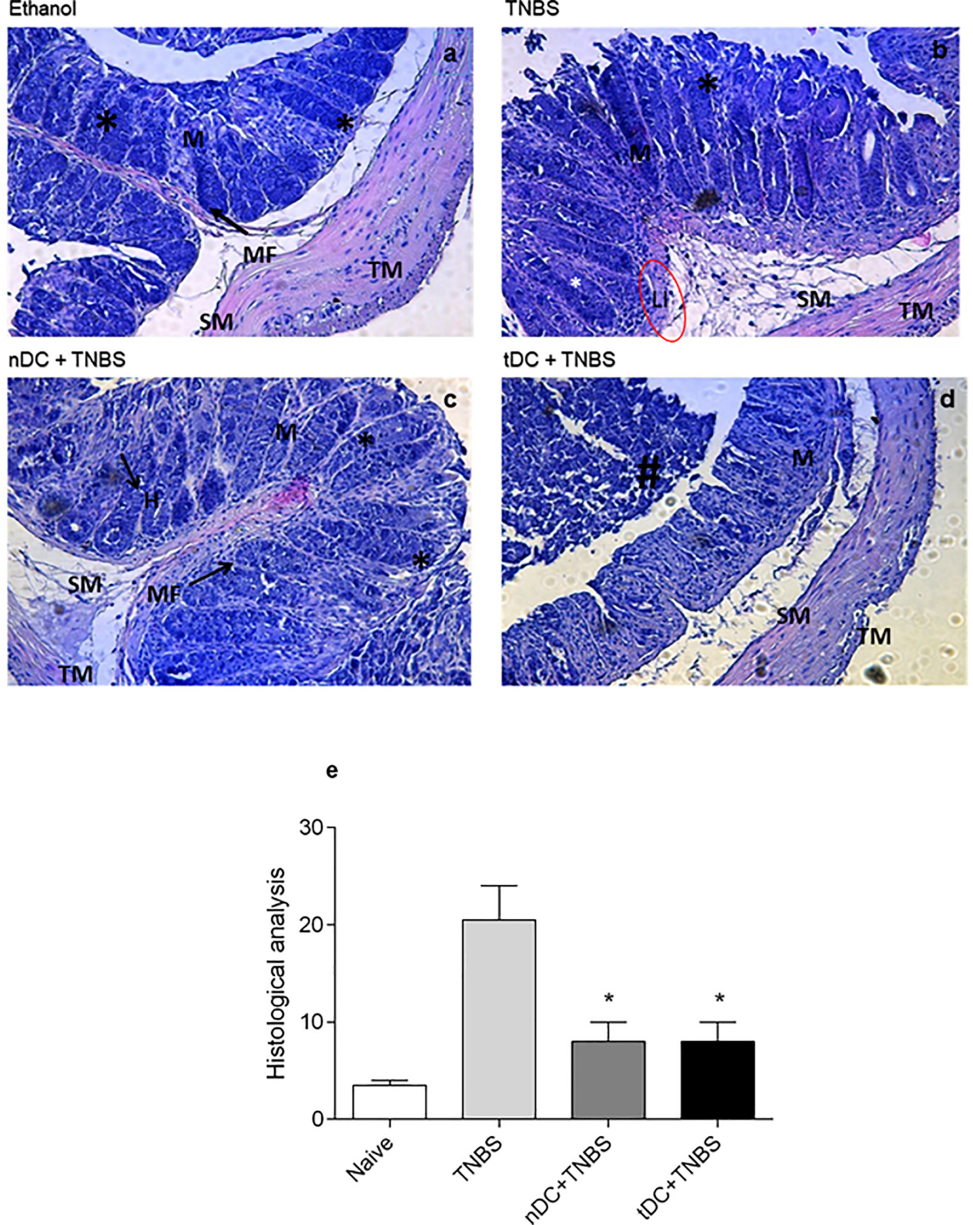
Transfer of DCs obtained from OVA-treated mice reduces histopathological damages in the large intestine caused by TNBS-induced colitis. BALB/c mice were exposed to the treatments described in Fig 1 and euthanized 5 days after induction of colitis. Distal portions of the large gut were collected and fixed in paraffin for histological processing. Panels: Ethanol, TNBS, nDC+TNBS, and tDC+TNBS represent sections obtained from mice treated with ethanol, TNBS, nDC, and TNBS or tDC and TNBS, respectively. Histological sections of 5μm were stained with hematoxylin and eosin and examined using light microscopy (200X).M: Tunica Mucosa, MF: mucosal folds, LI: lymphocytic infiltrate, TM: Tunica Muscularis, SM: Tunica Submucosa, *: Mucosal crypts, and #: Detached mucosal tissue debris.

As is widely known, colitis causes dramatic changes in the organization of the intestinal mucosa and submucosa. Fig 3B shows the deleterious effects caused by TNBS administration affecting mucosal folding and promoting an intense detachment of epithelial cells in the crypts of the intestinal lumen. Prior administration of CD11^+^ DCs from OVA-tolerant mice was, however, able to prevent tissue damage induced by TNBS (Fig 3D and 3F). Although administration of CD11^+^ DCs from naive mice did not completely prevent swelling, this treatment also significantly reduced tissue damage (Fig 3C and 3F). The protection against the effects of TNBS conferred by the treatment with DCs is similar to that which we observed in arthritis induced by injection of OVA emulsified in Freund's complete adjuvant. In that study, there was also a marked improvement in the clinical signs of the disease and reduction of the histopathological damages associated with osteoarthritis in DC-treated mice. Together with our previous results, the present study reinforces our hypothesis that the adoptive transfer of DC can result in considerable systemic benefits in inflammatory responses.

**Fig 4:**
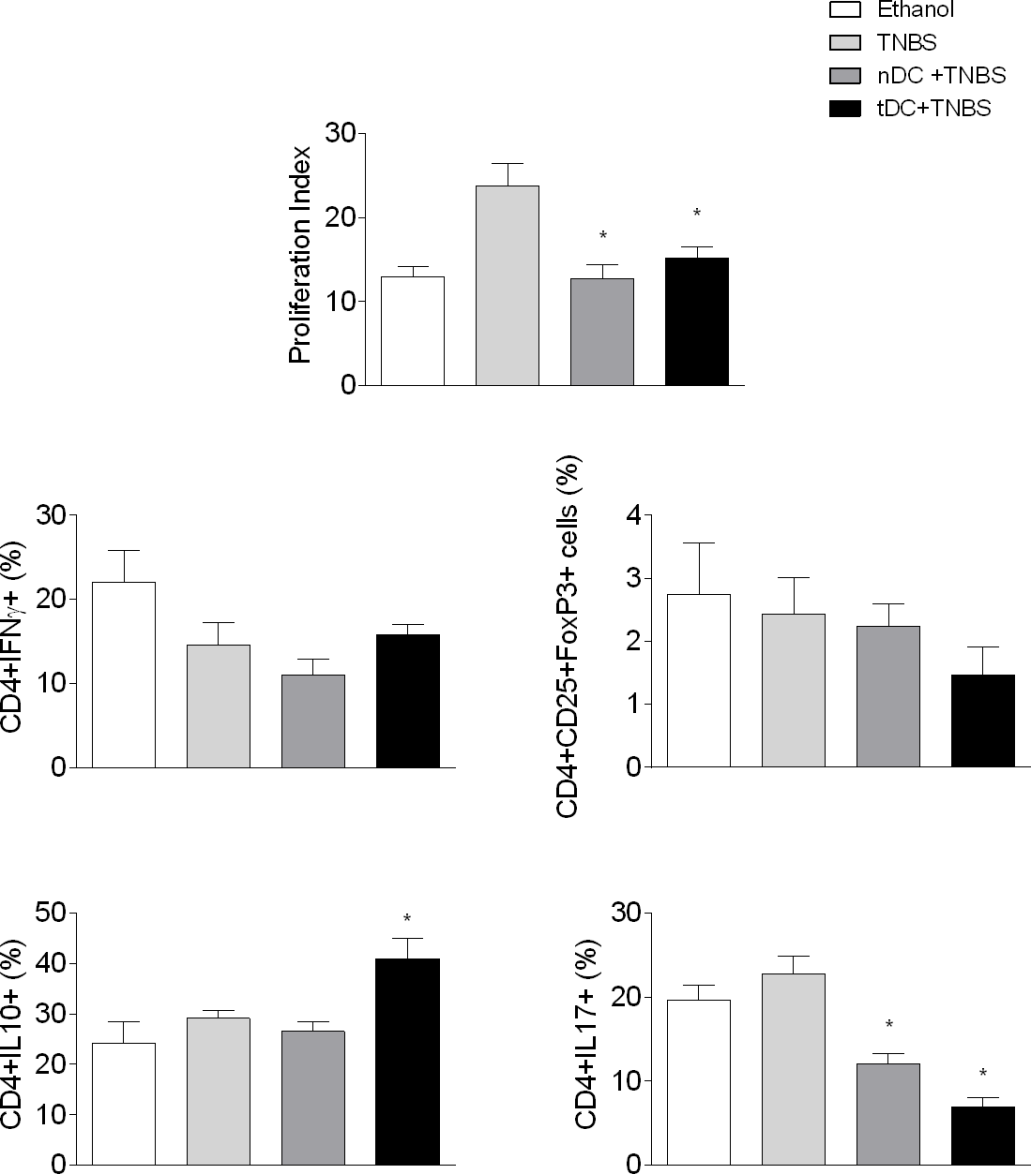
Adoptive transfer of CD11c^+^DCsalters the proliferative response, cytokine producing and transcription factor expressing-cells in cultures of spleen cells from mice with TNBS-induced colitis. Transfer of DCs and induction of colitis were performed as described in Fig 1. Mice were euthanized five days after TNBS instillation. Spleens were aseptically removed; cells were labeled with CFSE and cultured at a concentration of 2x10^6^ cells / ml in the presence of Concanavalin A (ConA; 2,5μg/ml) for 72 hours at 37°C and 5% CO2. Spleen cell proliferation (Panel A): cells were fixed in 1% formaldehyde and the readings performed by flow cytometer (FACSCalibur, BD). Proliferation was calculated using the software FCS Express and represents the inverse of the ratio of the fluorescence exhibited by the cells after 72 hours of culture and those immediately tested after labeling with CFSE. The cell frequency of T regulatory CD25^+^Foxp3^+^ cells (Panel B), IFN-γ (Panel C), IL-10 (Panel D), and IL-17 (Panel E) were monitored within the CD4^+^ T cell gate. The values correspond to the mean ± S.E.M. of the two independent experiments (n = 5). ANOVA followed by Bonferroni a posteriori test were used to determine statistical significance.

Since the adoptive transfer of CD11c DC^+^ significantly reduces the clinical signs and the local inflammatory manifestations of TNBS-induced colitis, we then sought to assess the effects of this treatment on the systemic immune responses of the treated mice. Initially, we investigated the proliferative responses of spleen cells cultured in the presence of Con-A. The spleen cells were labeled with CFSE and lectin-induced proliferation was assessed by flow cytometry. As shown in Fig 4, the proliferative responses of spleen cells obtained from DC-treated mice were similar to those observed in the cultures of naive spleen cells. However, their proliferative responses were significantly lower than those observed in the cultures of spleen cells collected from colitic control mice. Results similar to these had also been observed after the adoptive transfer of tDC in the experimental arthritis model [19].

After culturing with Con-A, the spleen cells were further labeled with specific antibodies to evaluate the expansion of T helper (Th1, Th2, and Th17) and Treg cells. Fig 4 shows that the frequency of Th17 cells was significantly reduced in the splenic cell cultures of mice treated with DC in comparison to the splenic cell cultures of colitic mice. No significant difference was found in the Treg cell frequency in the groups studied. However, the frequency of TCD4^+^ IL-10^+^ cells was higher in the cultures of spleen cells from the group treated with tDC than in the other groups. Although the release of IL-10 by T lymphocytes is important, other components of the immune response influencing its production, such as NKT and T γδ cells, cannot be excluded.

**Fig 5:**
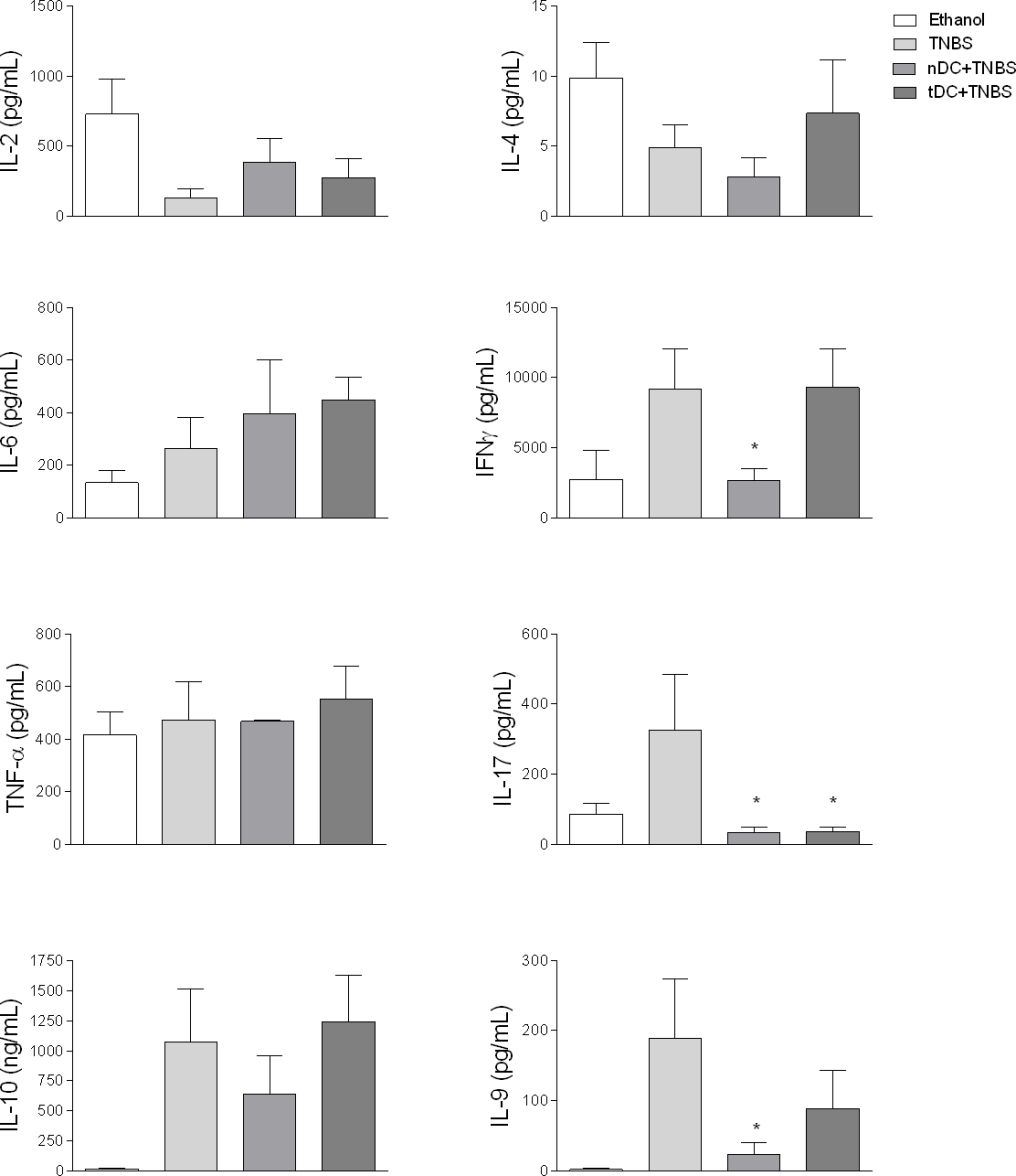
Adoptive transfer of DC alters cytokine levels in supernatants of ConA-stimulated spleen cells from mice with TNBS-induced colitis. Cultures of spleen cells were carried out as described in Fig 4. Cytokine levels were evaluated in the culture supernatants by using CBA Multiplex kit (Cytometric Bead Array Th1/Th2/Th17, BD Bioscience) and CBA kit (Cytometric Bead Array Th19, BD), and readings were performed by flow cytometer (FACSCalibur, BD). Cytokine concentrations were determined using the array FCAP TM Version 3.0 Software (BD). Results were expressed as means ± S.E.M. obtained from two independent experiments (n = 5). ANOVA followed by Bonferroni a posteriori test were used to determine statistical significance.

In addition, the production of cytokines measured in the supernatants of the splenic cell cultures was closely related to the variations observed in the different populations of TCD4^+^ cells examined here (Fig 5). Furthermore, it was possible to verify a significant reduction in IL-17 levels in supernatants of the splenic cell cultures of the tDC-treated mice compared to the control group. Although there was a slight increase in the levels of IL-4 and IL-10 in the cell cultures of the tDC-treated group, the results were not statistically significant. Significant variations were not observed in TNF-α levels among the different groups studied. A reduction was observed in IFN-γ levels in nDC-treated mice. These results resemble those obtained by our group in the collagen-OVA-induced arthritis model in BALB/c, thus indicating that the modulation of inflammatory disease by CD11c^+^DCs obtained from OVA-fed animals could be due to reduced production of IL-17 and frequency of Th17 cells, as well as an increase in IL-10^+^CD4^+^ cell frequency [19]. Just as we have postulated, other authors have also suggested that the augmented frequency of IL-10^+^CD4^+^ population plays an important role in the suppression of the exacerbated activation of inflammatory cytokines that result in inflammation and development of colitis [38, 39].

IL-9, a cytokine newly associated with IBD, may act as an inflammatory or regulatory cytokine because of its effects on both Th17 cells and Treg cells [40, 41]. It has been shown that IL-9 synergizes with TGF-beta in the differentiation of Th17 cells [19, 42–45]. On the other hand, Th17 cells can secrete IL-9 with regulatory effects. In vitro results confirm that IL-9 acts on Treg cells (CD4^+^FoxP3^+^ T cells), increasing its suppressor function through signaling pathways linked to the transcription factor STAT3 and STAT5 [46]. For this reason, IL-9 has been identified as a cytokine capable of initially inducing and then regulating tissue inflammation. These findings led us to investigate the presence of IL-9 in spleen cell culture supernatants from mice of the different experimental groups studied here. Our results show a clear trend of reducing IL-9 levels in spleen cell cultures collected from treated DCs (tDC and nDC) compared to the control group (Fig 5). Recent data has demonstrated a positive correlation between disease severity and IL-9 production in patients with Crohn’s disease, but not in patients with ulcerative colitis [41, 46, 47].

Although TNBS-induced colitis is considered a model for Crohn's disease, mimicking its clinical and inflammatory aspects, we observed in this study that the production of cytokines in the experimental disease, in particular IL-9, differs somewhat from that seen in the human disease. An earlier report showed that the adoptive transfer of tolerogenic DCs could modulate colitis induced by the inoculation of CD4^+^ CD25^+^ T cells (Treg CD4^+^ T cells) into SCID mice. The tolerogenic DCs employed in that study were differentiated from bone marrow cells cultured in the presence of IL-10 and pulsed with enteric bacteria extract, and their administration resulted in a significant reduction of the clinical and inflammatory signals associated with IBD in immunodeficient mice [7].

The results obtained in the present study corroborate previous data from our group on the ability of DC generated by induction of oral tolerance into a dietary protein [19]. The potential of CD11c^+^DCs obtained from tolerant animals in suppressing immune reactivity has also been demonstrated in other murine models such as allograft, autoimmune experimental encephalomyelitis (EAE), myasthenia gravis, and type 1 diabetes [48–50], which gives a greater degree of reliability to our data. Corroborating our data, Ohnmacht and collaborators demonstrated that mouse constitutively depleted of DC develop spontaneous autoimmune disease [51]. In addition, intravenous administration of CD11c^+^CD11b^+^ DC subset suppresses the symptoms of experimental autoimmune encephalomyelitis, a mouse model of multiple sclerosis (MS) [52, 53].

Although we had not evaluated the antigen-specific generation of T reg cells, Fukuya and colleagues demonstrated that CD11^+^DCs promote the transforming growth factor (TGF)-β1-mediated conversion of CD4^+^T cells into CD4^+^Foxp3^+^ induced T reg cells. They showed that, under steady-state conditions, the antigen-specific generation of Treg requires the function of CD11^+^DCs, whereas such a conversion was severely suppressed under inflammatory conditions [54]. However, it is already known that Tregs are phenotypically unstable and dysfunctional under inflammatory conditions [55]. Another study reports that IL-10 control of CD11c^+^ cells is essential to immune homeostasis in the intestine by limiting reactivation of local memory T cells and protecting against colitis, an alteration not found here [56]. Both treatment (nDC and tDC) reduced IL-17 and TCD4^+^IL17^+^ cell population. It is already known that Th17 cells play a crucial role in chronic intestinal inflammation. ROR gamma controls the IL-17 production that presents a highly pathogenic role in gut inflammation in the IBD model [57].

## Conclusion

Our results indicate that the adoptive transfer ofCD11c^+^cells obtained from naive or tolerant mice is able to partially reduce the immune response in TNBS-induced colitis. The immunomodulation observed can be attributed to the reduction in the number ofTh17 cells and secretion of IL-17 observed in both treatments.

Further studies are necessary to track and evaluate the *in vivo* effects of adoptive transfer of CD11c^+^DCs enrolled in this immune modulation. Thus, these cells may be a therapeutic alternative to modulate the immune responses in inflammatory disorders, specifically in human colitis.
